# Complete Genome Sequence of the Probiotic *Enterococcus faecalis* Symbioflor 1 Clone DSM 16431

**DOI:** 10.1128/genomeA.00165-12

**Published:** 2013-02-07

**Authors:** Moritz Fritzenwanker, Carsten Kuenne, Andre Billion, Torsten Hain, Kurt Zimmermann, Alexander Goesmann, Trinad Chakraborty, Eugen Domann

**Affiliations:** a Institute of Medical Microbiology, German Centre of Infection Research, Justus-Liebig-University, Giessen, Germany; b SymbioGruppe GmbH & Co KG, Herborn, Germany; c Institute for Bioinformatics, Center for Biotechnology (CeBiTec), Bielefeld University, Bielefeld, Germany

## Abstract

Here, we report the complete and annotated genome sequence of the probiotic *Enterococcus faecalis* Symbioflor 1 clone DSM 16431, included in a commercial probiotic product used for more than 50 years without any reports of infection. This sequence will provide new insights into the biology of this nonpathogenic and probiotic microorganism.

## GENOME ANNOUNCEMENT

Enterococci are facultative anaerobic Gram-positive cocci. They are regular commensals of the gastrointestinal tract, the oral cavity, and the vagina in humans (1). Enterococci can cause a wide variety of diseases in humans, including urinary tract infection, bacteremia, endocarditis, peritonitis, and wound infection (2, 3). Nevertheless, *Enterococcus faecalis* has potential benefits for human health and is currently used as food-starter cultures and probiotics. These organisms are used in traditional Mediterranean cheeses and other fermented foods such as sausages, olives, and vegetables. Furthermore, enterococci have been used as probiotics to improve the intestinal microbial balance (4, 5).

Here, we report the complete and annotated genome sequence of the nonpathogenic probiotic *E. faecalis* Symbioflor 1 clone DSM 16431. It was originally isolated in the 1950s from the stool specimen of a healthy human adult and has been in use as a probiotic for more than 50 years without any report or documentation of infection. Based on toxicological studies it has been shown that the strain is safe and can be used for direct human application (6, 7). The overall transcriptional responses of pathogenic *E. faecalis* strains and the Symbioflor 1 probiotic strain to growth in urine are highly conserved, suggesting that it is the presence or absence of virulence and adaptive traits rather than expression levels of these factors that determines pathogenic potential (8).

Three different kinds of libraries were prepared for sequencing. Initially, a genomic shotgun plasmid library (~2-kb inserts) was constructed and sequenced with Sanger sequencing technology. Subsequently, a standard library of sheared genomic DNA was sequenced on the GS FLX sequencer from Roche (Basel, Switzerland). A hybrid assembly with reads from both technologies (Sanger, 13,131 reads; 9.9 Mb; 454 pyrosequencing, 206,726 reads, 47.8 Mb) was created with the GS *de novo* assembler. Gaps between contigs were closed by PCR followed by Sanger sequencing. For larger gaps, a fosmid library (~40-kb inserts) (CopyControl fosmid library production kit, Epicenter, Madison, WI) was used as a template for genome closure. To further improve consensus quality, we also performed a sequencing run on the Illumina MiSeq system using a standard Nextera library, yielding 3,134,284 reads giving a total of 399.5 Mb (amounting to 164-fold coverage). The final assembly was conducted with MIRA version 3.9.4 (9), and DNASTAR SeqMan version 8.02 was employed to close remaining gaps. The genome comprises 2,810,675 bp and was annotated by using RAST (10) and GenDB (11). The circular genome contains 2,733 coding sequences and 63 tRNAs. The average GC content was determined to be 37.72%. Of note, Symbioflor 1 contains two major deletions in proximity to the *vanB*-associated island and the *efaB5* element, leading to the loss of *vanB* operon, bacteriocin, cytolysin L, enterococcal surface protein sp./*efaA*, gelatinase, and hyaluronidase genes (7, 12), thus providing important clues for its nonpathogenic nature. A unique region encoding a bacteriophage was detected at positions 1846700 through 1891973 by using PHAST (13).

### Nucleotide sequence accession number.

The genome sequence of *E. faecalis* Symbioflor 1 clone DSM 16431 has been deposited in the EMBL database under accession number HF558530.

## References

[1] Teixeira LM Carvalho Mda G Facklam RR 2007 Enterococcus, p 430–442 In Baron EJ Murray PR Jorgensen JH Landry ML Pfaller MA Manual of clinical microbiology, 9th ed ASM Press, Washington, DC

[2] Hunt CP 1998 The emergence of enterococci as a cause of nosocomial infection. Br. J. Biomed. Sci. 55:149–156 10198473

[3] Jett BD Huycke MM Gilmore MS 1994 Virulence of enterococci. Clin. Microbiol. Rev. 7:462–478 7834601 10.1128/cmr.7.4.462 PMC358337

[4] Marteau P Seksik P Lepage P Doré J 2004 Cellular and physiological effects of probiotics and prebiotics. Mini Rev. Med. Chem. 4:889–896 15544550 10.2174/1389557043403369

[5] Ouwehand AC Salminen S Isolauri E 2002 Probiotics: an overview of beneficial effects. Antonie Van Leeuwenhoek 82:279–289 12369194

[6] Christoffersen TE Jensen H Kleiveland CR Dørum G Jacobsen M Lea T 2012 In vitro comparison of commensal, probiotic and pathogenic strains of *Enterococcus faecalis*. Br. J. Nutr. 108:2043–2053 22348541 10.1017/S0007114512000220

[7] Domann E Hain T Ghai R Billion A Kuenne C Zimmermann K Chakraborty T 2007 Comparative genomic analysis for the presence of potential enterococcal virulence factors in the probiotic *Enterococcus faecalis* strain Symbioflor 1. Int. J. Med. Microbiol. 297:533–539 17466591 10.1016/j.ijmm.2007.02.008

[8] Vebø HC Solheim M Snipen L Nes IF Brede DA 2010 Comparative genomic analysis of pathogenic and probiotic *Enterococcus faecalis* isolates, and their transcriptional responses to growth in human urine. PLoS One 5:e12489 20824220 10.1371/journal.pone.0012489 PMC2930860

[9] Chevreux B Wetter T Suhai S 1999 Genome sequence assembly using trace signals and additional sequence information, p 45–56 In Computer science and biology: proceedings of the German conference on bioinformatics (GCB)99, Hannover, Germany.

[10] Aziz RK Bartels D Best AA DeJongh M Disz T Edwards RA Formsma K Gerdes S Glass EM Kubal M Meyer F Olsen GJ Olson R Osterman AL Overbeek RA McNeil LK Paarmann D Paczian T Parrello B Pusch GD Reich C Stevens R Vassieva O Vonstein V Wilke A Zagnitko O 2008 The RAST server: rapid annotations using subsystems technology. BMC Genomics 9:75 18261238 10.1186/1471-2164-9-75 PMC2265698

[11] Meyer F Goesmann A McHardy AC Bartels D Bekel T Clausen J Kalinowski J Linke B Rupp O Giegerich R Pühler A 2003 GenDB: an open source genome annotation system for prokaryote genomes. Nucleic Acids Res. 31:2187–2195 12682369 10.1093/nar/gkg312 PMC153740

[12] Paulsen IT Banerjei L Myers GS Nelson KE Seshadri R Read TD Fouts DE Eisen JA Gill SR Heidelberg JF Tettelin H Dodson RJ Umayam L Brinkac L Beanan M Daugherty S DeBoy RT Durkin S Kolonay J Madupu R Nelson W Vamathevan J Tran B Upton J Hansen T Shetty J Khouri H Utterback T Radune D Ketchum KA Dougherty BA Fraser CM 2003 Role of mobile DNA in the evolution of vancomycin-resistant *Enterococcus faecalis*. Science 299:2071–2074 12663927 10.1126/science.1080613

[13] Zhou Y Liang Y Lynch KH Dennis JJ Wishart DS 2011 PHAST: a fast phage search tool. Nucleic Acids Res. 39:W347–W352 21672955 10.1093/nar/gkr485 PMC3125810

